# Two new species of *Aristocleidus* (Monogenea) from the gills of the Mexican mojarra *Eugerres mexicanus* (Perciformes, Gerreidae) from southwestern Mexico

**DOI:** 10.1051/parasite/2015033

**Published:** 2015-11-25

**Authors:** Edgar F. Mendoza-Franco, Marina Tapia Osorio, Juan Manuel Caspeta-Mandujano

**Affiliations:** 1 Instituto de Ecología, Pesquerías y Oceanografía del Golfo de México (EPOMEX), Av. Héroe de Nacozari No. 480, CP. 24029, Universidad Autónoma de Campeche San Francisco de Campeche Campeche Mexico; 2 Facultad de Ciencias Biológicas y Centro de Investigaciones Biológicas, Laboratorio de Parasitología de Animales Silvestres, Universidad Autónoma del Estado de Morelos, Avenida Universidad No. 1001, Colonia Chamilpa 62209 Cuernavaca Morelos Mexico

**Keywords:** *Aristocleidus*, Dactylogyridae, *Eugerres mexicanus*, Chiapas, Mexico, Gerreidae, Moronidae

## Abstract

*Aristocleidus mexicanus* n. sp. and *Aristocleidus lacantuni* n. sp. are described from the gills of the Mexican mojarra *Eugerres mexicanus* (Gerreidae, Perciformes) from the Rio Lacantún basin, Chiapas State, Mexico. These new species differ from previously described congeneric species in the characteristics of several structures, including: (a) ventral anchors, with differences in length (i.e. 46–50 µm in *A. mexicanus* vs. 38–43 µm, 34–37 µm, and 26–33 µm in *Aristocleidus hastatus* Mueller, 1936, *Aristocleidus* sp. of Mendoza-Franco, Violante-González & Roche 2009, and *Aristocleidus lamothei* Kritsky & Mendoza-Franco, 2008, respectively) and shape (i.e. slightly angular union of elongate arcing shaft and point in *A. mexicanus* vs. point and shaft united at a conspicuous angular bend in *A. hastatus* and *Aristocleidus* sp., and evenly curved shaft and point in *A. lamothei*); (b) male copulatory organ, i.e. a coiled tube with less than one ring in *A. mexicanus* and *A. lacantuni* (vs. a coiled tube of about 1½ in *Aristocleidus* sp.); (c) distal end of the accessory piece (ornate in *A. mexicanus* vs. distally flattened and trifid in *A. hastatus* and *A. lamothei*, respectively); (d) vaginal tube (moderately long in *A. mexicanus* vs. short in *A. lamothei* and looping in *Aristocleidus* sp.); and (e) ventral bar (anteromedial process with terminal horn-like ornamentation in *A. lacantuni* vs. ornamentation absent in the other species). This study reports for the first time species of *Aristocleidus* from freshwater environments in Mexico.

## Abstract (fr)

*Aristocleidus mexicanus* n. sp. et *Aristocleidus lacantuni* n. sp. sont décrits des branchies du poisson *Eugerres mexicanus* (Gerreidae, Perciformes) du bassin du Rio Lacantun, État du Chiapas, Mexique. Ces nouvelles espèces diffèrent des espèces congénériques décrites précédemment par différentes structures : (a) anchors ventraux, avec des différences de longueur (soit 46-50 µm chez *A. mexicanus* contre respectivement 38-43 µm, 34-37 µm, et 26-33 µm chez *Aristocleidus hastatus* Mueller, 1936, *Aristocleidus* sp. de Mendoza-Franco, Violante-González & Roche, 2009 et *Aristocleidus lamothei* Kritsky & Mendoza-Franco, 2008) et de forme (c’est-à-dire une union légèrement angulaire du manche allongé et de la pointe chez *A. mexicanus*, contre une pointe et un manche unis avec une courbe bien visible chez *A. hastatus* et *Aristocleidus* sp. et un manche régulièrement courbe chez *A. lamothei*) ; (b) organe copulateur mâle (tube enroulé avec moins de 1 tour chez *A. mexicanus* et *A. lacantuni* (contre un tube enroulé d'environ 1½ tour chez *Aristocleidus* sp.) ; (c) extrémité distale de la pièce accessoire (ornée chez *A. mexicanus* contre distalement aplatie et trifide chez *A. hastatus* et *A. lamothei*, respectivement) ; (d) tube vaginal (modérément long chez *A. mexicanus* contre court chez *A. lamothei* et en boucle chez *Aristocleidus* sp.) ; et (e) barre ventrale (processus antéromédian avec ornementation terminale en forme de corne chez *A. lacantuni* contre ornementation absente chez les autres espèces). Cette étude rapporte pour la première fois des espèces d’*Aristocleidus* en eau douce au Mexique.

## Introduction

Gerreidae comprises a group of marine and/or brackish water fish commonly known as mojarras distributed along the subtropical and tropical coastal areas of the world. In the Americas, Gerreidae consists of 22 valid species belonging to four genera: *Gerres* Quoy & Gaimard, *Diapterus* Ranzani, *Eucinostomus* Baird & Girard, and *Eugerres* Jordan & Evermann [2]. *Eugerres* includes seven species from which only *Eugerres mexicanus* (Steindachner, 1863) and *Eugerres castroaguirrei* González-Acosta & Rodiles-Hernández, 2013 are confined to freshwater habitats in southeastern Mexico and northern Guatemala [2–4]. *Diapterus auratus* Ranzani, 1842, *Diapterus peruvianus* (Cuvier), *Diapterus rhombeus* (Cuvier), *Eugerres brasilianus* (Cuvier), *Eugerres plumieri* (Cuvier), and *Gerres cinereus* Jordan & Evermann of the Gulf (coastal areas from the States of Veracruz and Yucatán) and Pacific (a coastal lagoon from the state of Guerrero) of Mexico and Panama are known to be parasitized by species of *Aristocleidus* Mueller, 1936 (Dactylogyridae), i.e., *A. hastatus* (type species of the genus), *Aristocleidus lamothei* Kritsky & Mendoza-Franco, 2008, and *Aristocleidus* sp. of Mendoza-Franco et al. [8, 11]. On the other hand, only *A. hastatus* has been described from the gills of the striped bass, *Roccus lineatus* (Bloch) [now *Morone saxatilis* (Walbaum)] (Moronidae) in the Peace River near Fort Ogden, Florida [16]. During investigations into the helminth fauna of fishes from the Rio Lacantún basin in the state of Chiapas (Mexican Pacific), two undescribed species of *Aristocleidus* were found on the gills of the Mexican mojarra *E. mexicanus* in January and August 2014. These new species are described in this article.

## Materials and methods

Host specimens of *E. mexicanus* were captured by hook-and-line and throw nets in January and August 2014 in the Rio Lacantún basin in the state of Chiapas, Mexico (16°09′96.6″ N, 90°95′56.9″ W). Live fish were sacrificed bloodlessly by puncturing the brain region (a needle is introduced dorsally via the eye socket and moved about to destroy the spinal cord) [13]. The gills of each fish were removed and placed in finger bowls containing 4–5% formalin solution to fix any of the ectoparasites that might be present. Subsequently, parasites were isolated and stained with Gomori’s trichrome and mounted in Canada balsam. In addition, some specimens were mounted with a mixture of acid-lactic (AL) and glycerin-ammonium picrate [12] to obtain measurements and line drawings of haptoral structures and the copulatory complex. All other measurements were obtained from unflattened specimens stained with Gomori’s trichrome. Drawings were made with the aid of a drawing tube using a Leica microscope DM50 with Nomarski interference contrast. Measurements, all in micrometers, represent straight-line distances between extreme points and are expressed as the mean followed by the range and number (*n*) of structures measured in parentheses; body length includes that of the haptor; measurements of the copulatory complex, anchor/base are represented in Figures 2 and 3 as the perpendicular distance between parallel lines. Direction of the coil of the copulatory organ, i.e., clockwise, was determined using the procedure suggested by Kritsky et al. [7]. Haptoral terminology for species of *Aristocleidus* is that provided by Kritsky & Mendoza-Franco [8]. Type specimens are deposited in the National Helminthological Collection of Mexico (CNHE), Institute of Biology, National Autonomous University of Mexico, Mexico.

**Figures 1–8.**
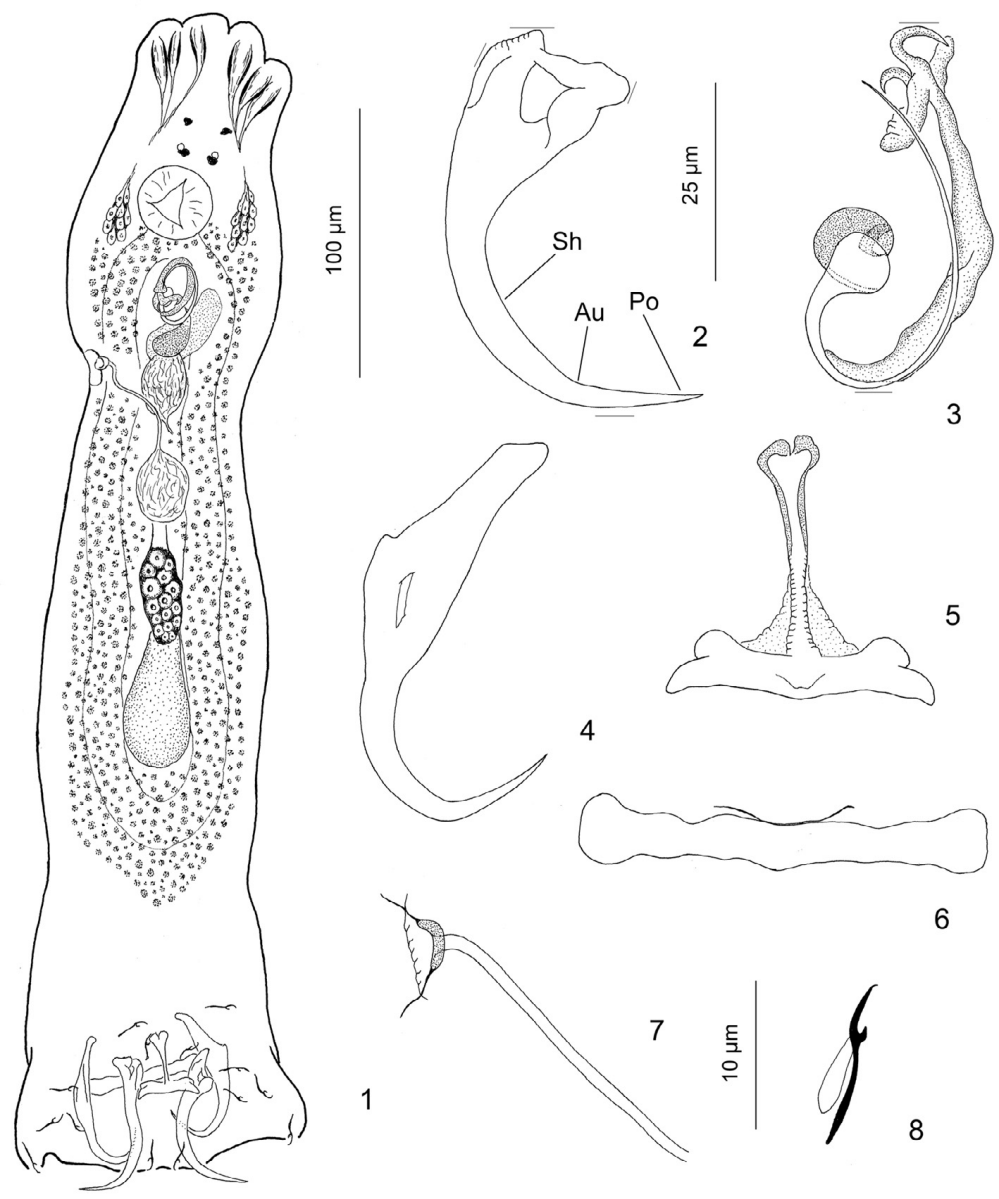
*Aristocleidus mexicanus* n. sp. from the gills of *Eugerres mexicanus*. 1: whole-mount (composite, ventral view); 2: ventral anchor (Sh, shaft; Au, angular union; Po, point); 3: copulatory complex (dorsal view); 4: dorsal anchor; 5: Ventral bar; 6: dorsal bar; 7: vagina; 8: hook. Figures are drawn to the following scales: 100 μm (Fig. 1), 25 μm (Figs. 2–7), and 10 μm (Fig. 8).

## Results

### *Aristocleidus mexicanus* n. sp.

urn:lsid:zoobank.org:act:8CE4BC57-1863-4101-9A8D-409B5A1476D3

Type-host: *Eugerres mexicanus* (Steindachner, 1863) (Perciformes, Gerreidae).

Site of infection: Gill lamellae.

Type locality and collection date: Rio Lacantún basin (16°09′96.6″ N, 90°95′56.9″ W), August 2014.

Specimens deposited: holotype, 12 paratypes and 6 vouchers in CNHE (9872, 9873, and 9874, respectively).

Etymology: named after its host.

#### Description (Figs. 1–8)

Diagnosis (based on 13 specimens collected in August and on 6 collected in January [brackets]) includes the following: Body 515 (425–630; *n* = 10) long; greatest width 118 (85–150; *n* = 10) usually near gonads. Cephalic lobes well developed; each head organ comprising groupings of terminations of cephalic-gland ducts. Pharynx 35 (25–45; *n* = 10) wide, subspherical. Testis 30–40 (*n* = 2) long, 18 (17–20; *n* = 3) wide, pyriform; seminal vesicle comparatively large, fusiform, lying in the midline of body; prostatic reservoir pyriform. Copulatory complex 39 (27–50; *n* = 12) [47 (43–51; *n* = 5)] long. Male copulatory organ (MCO) comprising proximal funnel-shaped base with proximal thickened edge, coiled tube of less than one ring; J-shaped accessory piece, terminally ornate. Prostatic glands conspicuous surrounding dorsal region of prostatic reservoir. Germarium 54 (35–75; *n* = 6) long, 19 (15–25; *n* = 5) wide; vaginal vestibule thick walled, internal, communicating with vaginal pore by short duct; vaginal tube moderately long, delicate, extending to ovate seminal receptacle; vitellarium dense, vitelline ducts not observed. Haptor 102 (90–120; *n* = 9) wide. Ventral anchor 48 (46–50; *n* = 22) [50 (43–57; *n* = 8)] long, with variably bent roots, slightly angular union of elongate arcing shaft and straight point (see Au, Sh and Po in Fig. 2); base 15 (13–18; *n* = 17) [18 (16–20; *n* = 8)]. Dorsal anchor 48 (43–51; *n* = 15) [51 (48–57; *n* = 11)] long, with elongate superficial root, short to non-existent deep root, slightly arced shaft, elongate recurved point. Ventral bar 26 (23–30; *n* = 12) [33 (31–35; *n* = 3)] long, bilaterally flattened with well-differentiated anteromedial process which is basally covered by a vellum-like fine tissue, a concavity divided into two lateral rounded expansions distally. Dorsal bar 45 (42–47; *n* = 13) [57 (50–67; *n* = 3)] long, with slightly expanded ends, indistinct saucer-shaped flange closely appressed to bar proper. Hook 12 (12–13; *n* = 14) [12 (11–13; *n* = 14)] long; filamentous hooklet (FH) loop about 70% shank length.

**Figures 9–14.**
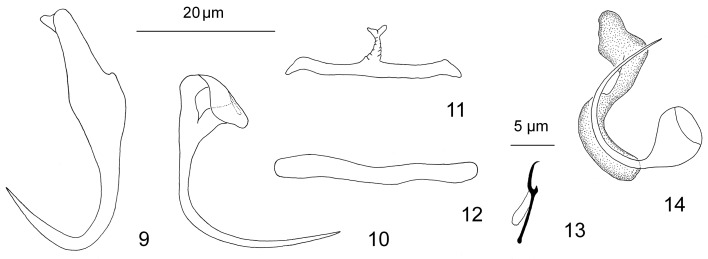
Haptoral and copulatory complex sclerites of *Aristocleidus lacantuni* n. sp. from the gills of *Eugerres mexicanus*. 9: dorsal anchor; 10: ventral anchor; 11: ventral bar; 12: dorsal bar; 13: hook; 14: Copulatory complex. Figures are drawn to the following scales: 20 μm (Figs. 9–12 and 14) and 5 μm (Fig. 13).

#### Differential diagnosis

Placement of this new species in *Aristocleidus* is based on agreement with the amended generic diagnosis provided by Kritsky and Mendoza-Franco [8], i.e., species with gonads overlapping, ventral anchors with deeply incised base forming deep and superficial roots, a coiled tube of the MCO (non-articulated with the accessory piece) in a clockwise orientation, and haptoral hooks with upright acute thumb, slender shank comprising one subunit and FH loop. *A. mexicanus* n. sp. most resembles *A. hastatus* and *Aristocleidus* sp. from *Gerres cinereus* from Chautengo Lagoon from Guerrero State (Mexican Pacific) in having ventral bar with conspicuous anteromedial process and by the general morphology of the vaginal tube. *A. mexicanus* n. sp. differs from these two latter species and *A. lamothei* mainly in the length (i.e., 46–50 µm vs. 38–43, 34–37, and 26–33, respectively) and shape (i.e., slightly angular union of elongate arcing shaft and point [see Au, Sh and Po, respectively, in Fig. 2] vs. point and shaft united at a conspicuous angular bend in *A. hastatus* and *Aristocleidus* sp. and evenly curved shaft and point in *A. lamothei*) of their ventral anchors and by having a coiled tube of the MCO with less than one ring (a coiled tube of about 1½ in *Aristocleidus* sp.), a vaginal tube moderately long (short vaginal tube in *A. lamothei* and looping in *Aristocleidus* sp.), and an ornate distal end of the accessory piece (distally flattened and trifid accessory piece in *A. hastatus* and *A. lamothei*, respectively) [8, 11].

### *Aristocleidus lacantuni* n. sp.

urn:lsid:zoobank.org:act:6A4D7AD9-B9D8-48E7-85FF-37A182476C2D

Type-host: *Eugerres mexicanus* (Steindachner, 1863) (Perciformes, Gerridae).

Site of infection: Gill lamellae.

Type locality and collection date: Rio Lacantún basin (16°09′96.6″ N, 90°95′56.9″ W), August 2014.

Specimens deposited: holotype, three paratypes in CNHE (9875 and 9876, respectively).

Etymology: named after the location (i.e., Rio Lacantún) from which this species was found.

#### Description (Figs. 9–14)

Diagnosis (based on four specimens) includes the following: Body 302–342 long; greatest width 90–102 usually near body midlength. Cephalic lobes well developed; each head organ comprising groupings of terminations of cephalic-gland ducts. Pharynx 27 wide, subspherical to elongate ovate. Testis ovate; seminal vesicle comparatively large, fusiform; prostatic reservoir pyriform. Copulatory complex 27 (23–30; *n* = 4) long. MCO comprising proximal funnel-shaped base, coiled tube loose, comprising about 0.5 poorly defined ring, appearing U-shaped; poorly defined Z-shaped accessory piece. Germarium 44–48 long, 16 wide; vaginal tube short, opening on left side of the body, poorly sclerotized (not depicted); vitellarium dense, vitelline ducts not observed. Haptor 55–58 wide. Ventral anchor 26 (25–28; *n* = 7) long, with straight shaft and elongate arcing point, base with fused roots, ventrally bent; base 10 (9–11; *n* = 7). Dorsal anchor 36 (35–38; *n* = 7) long, with elongate superficial root with a small distal knob-like extension, short to non-existent deep root, slightly arced shaft, elongate recurved point. Ventral bar 26 (24–28; *n* = 3) long, with conspicuous anteromedial process with terminal horn-like ornamentation; dorsal bar 25 (22–29; *n* = 3) long, straight, rod-shaped. Hook 11 (10–13; *n* = 10) long; FH loop about 60% shank length.

#### Differential diagnosis

The morphology of the haptoral and copulatory sclerites of this species clearly allows its formal generic assignment within *Aristocleidus*. *A. lacantuni* n. sp. resembles *A. hastatus* and *A. lamothei* in the length of the copulatory complex, i.e., 23–30 vs. 27–36 and 22–26, respectively. It differs from these latter species as well as *A. mexicanus* n. sp. and *Aristocleidus* sp. in having shorter anteromedial process of the ventral bar (comparatively larger in *A. hastatus*, *A. mexicanus* n. sp., and *Aristocleidus* sp. and indistinct in *A. lamothei*) and a ventral anchor with straight shaft and elongate arcing point (point and shaft united at a conspicuous angular bend in *A. hastatus* and *Aristocleidus* sp., evenly curved shaft and point in *A. lamothei*, and elongate arcing shaft and straight point [slightly angular union] in *A. mexicanus* n. sp.) [8, 11].

## Discussion

The diagnostic characters used previously to differentiate species of *Aristocleidus* involved the morphology of the haptoral armament (i.e., shape of the connection between shaft and point of anchors) and copulatory complex (i.e., shape of the distal end of the accessory) [8, 11]. This study showed that these characters are reliable for separation of *A. mexicanus* n. sp. and *A. lacantuni* n. sp. The occurrence of species of *Aristocleidus* on primarily marine fish (i.e., species of *Diapterus*) has generated the hypothesis that these parasite species originated in the marine environment [8]. For example, *A. hastatus* and *A. lamothei* have been reported from *Eugerres plumieri*, *Diapterus auratus*, and *Diapterus rhombeus* from rivers (i.e., Rio Maquinas in Veracruz State) and/or estuarine systems (i.e., Ria Celestún in Yucatán State) draining to the Gulf of Mexico. Similarly, on the Pacific coast of Mexico and Panama, these monogeneans as well as *Aristocleidus* sp. have been reported from *Diapterus peruvianus* and *Gerres cinereus* from the Chautengo and Tres Palos Lagoons in Guerrero State and *Eugerres brasilianus* from Gatun Lake in Panama [8, 11].

However, this study demonstrates that *A. mexicanus* n. sp. and *A. lacantuni* n. sp. in *Eugerres mexicanus* are clearly secondary invaders of freshwater. *E. mexicanus* inhabit freshwater habitats along the rivers Grijalva-Usumacinta and Coatzacoalcos basins in southeastern Mexico (Chiapas, Tabasco, and Veracruz) and northern Guatemala [14, 15], with a noteworthy distribution in highlands with elevations of 100–300 m [4] (i.e., Río Lacantún basin) and the northern part of Guatemala (upper Usumacinta). Thus, a marine ancestor of these monogeneans along with their hosts could have colonized this freshwater environment from which they speciated through adaptive processes (evolutionary radiation of fish in this region indicates old ichthyofauna dated from the late Cretaceous [60 Mya]) [1, 10]. To date, *A. mexicanus* n. sp. and *A. lacantuni* n. sp. appear to be limited or dominant under freshwater conditions, while their euryhaline or marine congeneric species are absent or uncommon. In fact, there are other examples of tropical marine species of monogeneans that have secondarily invaded freshwater, i.e., species of *Euryhaliotrema* Kritsky & Boeger, 2002 (Dactylogyridae) and *Diplectanum* Diesing, 1858 (Diplectanidae) from South American freshwater croakers, *Plagioscion* spp. (Sciaenidae) [6, 9]. Examination of other freshwater gerreids in the Rio Lacantún basin (i.e., *Eugerres castroaguirrei* González-Acosta & Rodiles-Hernández 2013) would help to clarify the rule of the freshwater vs. saltwater on the presence or absence of species of *Aristocleidus*. For example, salinity has been reported to be a determining factor for the occurrence of species of *Rhabdosynochus* (Diplectanidae) on snooks, *Centropomus* spp. (Centropomidae) in Florida [5].

Knowledge about the phylogenetic affinities between gerreids and the moronid type host (i.e., *M. saxatilis*) of the first described species of *Aristocleidus* (i.e., *A. hastatus*) is unknown. Chen et al. [2] provided a molecular phylogenetic hypothesis using four genera (*D. auratus* plus *E. plumieri* and *Eucinostomus gula* [Quoy & Gaimard] plus *G. cinereus*) of the Gerreidae including other percomorph fishes and stated that this latter host family is monophyletic. In that hypothesis, gerreids are placed at an intermediate position in the percomorph tree between early branching lineages such as scombrids (i.e., *Scomberomorus commerson* [Lacépède]), serranids (i.e., *Holanthias chrysostictus* [Günther]), and a terminal clade containing tetraodontiforms (i.e., *Takifugu rubripes* [Temminck & Schlegel]), Lutjanids (i.e., *Lutjanus analis* [Cuvier]), and other percoid relatives such as north American moronids, i.e., *Dicentrarchus labrax* (Linnaeus). These gerreids within the phylogenetic hypothesis of Chen et al. [2] suggest that this gerreid group is comparatively old and predates the origins of the Moronidae. If this were true, then, the occurrence of *A. hastatus* on *M. saxatilis* apparently took place comparatively recently and probably corresponds to the concomitant colonization of an anadromous common ancestor to this host family that gave rise to the north American estuarine and freshwater species [17]. It is clear that further surveys of species of *Aristocleidus* infesting gerreids (and probably moronids) from the western Atlantic and the eastern Pacific will be necessary to understand the diversification of *Aristocleidus* spp. in the tropical areas.
